# Recent trends and advances in single-atom nanozymes for the electrochemical and optical sensing of pesticide residues in food and water

**DOI:** 10.1039/d5ra00474h

**Published:** 2025-05-14

**Authors:** Raed Obaid Saleh, Ebraheem Abdu Musad Saleh, M. M. Moharam, Subasini Uthirapathy, Suhas Ballal, Abhayveer Singh, Anima Nanda, Subhashree Ray, Abdul_Kareem Nasir, Rzaq Shailaan Kaurshead

**Affiliations:** a Department of Medical Laboratories Techniques, College of Health and Medical Techniques, University of Al Maarif Al Anbar 31001 Iraq; b Department of Chemistry, College of Science and Humanities in Al-Kharj, Prince Sattam bin Abdulaziz University Al-Kharj 11942 Saudi Arabia e.saleh@psau.edu.sa; c Chemical and Electrochemical Processing Department, Central Metallurgical Research and Development Institute (CMRDI) P. O. Box 87, Helwan 11421 Egypt; d Pharmacy Department, Tishk International University Erbil Kurdistan Region Iraq subasini.uthirapathy@tiu.edu.iq; e Department of Chemistry and Biochemistry, School of Sciences, JAIN (Deemed to be University) Bangalore Karnataka India; f Centre for Research Impact & Outcome, Chitkara University Institute of Engineering and Technology, Chitkara University Rajpura 140401 Punjab India; g Department of Biomedical, Sathyabama Institute of Science and Technology Chennai Tamil Nadu India; h Department of Biochemistry IMS and SUM Hospital, Siksha ‘O’ Anusandhan (Deemed to be University) Bhubaneswar Odisha-751003 India; i Mazaya University College Dhiqar Iraq; j Laboratories Techniques Department, College of Health and Medical Techniques, Al-Mustaqbal University 51001 Babylon Iraq

## Abstract

Nowadays, single-atom nanozymes (SAzymes) and single-atom catalysts (SACs) have flourished in the field of catalysis owing to their high catalytic performance and exceptional atom utilization efficiency, thereby enhancing biosensing capabilities. In comparison to natural enzymes, SAzymes offer several advantages, including cost-effectiveness, ease of production, and robust catalytic activity, making them highly promising for biosensing applications. Notably, SAzymes demonstrate superior catalytic efficiency and selectivity compared with traditional nanozymes. In this context, this review delineates the enzyme-like characteristics of SAzymes aimed at enhancing food safety, with a focus on the primary factors that influence their catalytic efficacy. The discussion has been expanded to include the use of SAzymes for screening various pesticide residues, particularly organophosphate pesticides (OPPs), carbamates, acetamiprid, pyrethroids, and other pesticide types, which are present in agricultural food products. These applications are realized because of the exceptional properties of single-atom structures, including enhanced reaction kinetics, high active site density, and tunable electronic properties. The integration of SAzymes into sensing platforms holds great potential for the development of cost-effective, sensitive, and reliable tools for the real-time monitoring of pesticide residues. Finally, this paper highlights the current challenges and outlines potential opportunities for the advancement of SAzyme-based biosensing technologies.

## Abstract 2

Nowadays, single-atom nanozymes (SAzymes) and single-atom catalysts (SACs) have flourished in the field of catalysis owing to their high catalytic performance and exceptional atom utilization efficiency, thereby enhancing biosensing capabilities.

## Graphical abstract

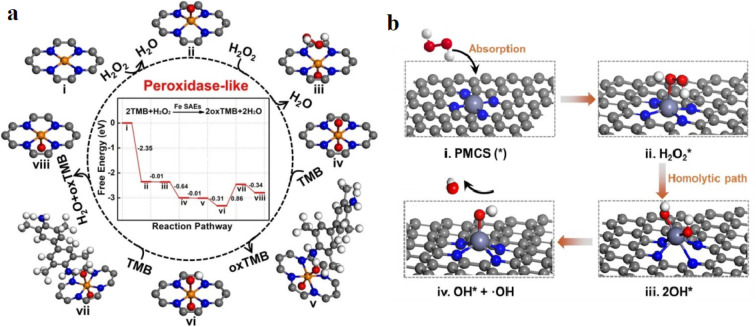

## 1. Introduction

Pesticides are chemical or organic substances or their combinations that are employed to manage, eradicate, prevent, regulate, or repel illnesses, pests, insects, and weeds that impact the growth of plants.^1,2^ These substances are frequently categorized according to their chemical composition, method of application, hazard level, mode of action, and timing of application. Crop yields in agriculture have significantly increased in recent decades because of the use of chemical pesticides. The global demand for pesticides has steadily risen since the mid-1940s, primarily as a result of the expansion of commercial agriculture. According to the FAO, the global use of pesticides has steadily increased, reaching a market value of $84.5 billion in 2019, with a CAGR of 4.2% since 2015. However, pesticide contamination has an adverse effect on both the human health and environment; in particular, it is becoming a major concern when it comes to vegetables and fruits, as the world is increasingly shifting to healthy eating habits. The uncontrolled overuse of pesticides comprising chlorpyrifos, glyphosate, pyrethroids, mancozeb, and neonicotinoids in the production of vegetables and fruits has adversely affected the health of people.^3^ The side effects include lightheadedness, headaches, nausea, rashes, vomiting, and more serious consequences linked to the nervous system, endocrine system, and immune system. Additionally, prolonged or repeated exposure to specific pesticides can result in long-term health issues, such as infertility, cancer, and neurological disorders.^4^ Furthermore, from an environmental perspective, pesticides present a significant risk to ecosystems as they have the potential to disrupt the intricate balance of these systems and accidently affect non-target species, including mammals and beneficial insects. The presence of pesticide residues in water and soil poses significant risks to aquatic organisms and soil ecosystems, thereby threatening the overall ecological equilibrium.^5^ Consumers face significant risks owing to the accumulation of harmful residues in food products, which is a consequence of the negligent use of pesticides in agricultural activities. Therefore, there is a pressing requirement for convenient and effective techniques for screening chemical pesticide residues.

Biosensor techniques are very valuable tools for pesticide residue analysis. A biosensor is a diagnostic tool that combines a biological recognition factor (*e.g.*, enzymes, antibodies, or nucleic acids) and a physicochemical transducer to identify the existence of specific analytes.^6^ Among various biosensor systems, nanozyme-based optical and electrochemical sensors have attracted increasing interest. Nowadays, nanozymes are increasingly recognized as viable alternatives to natural enzymes owing to their numerous enzyme-mimicking properties, ease of mass production, cost-effective synthesis, excellent stability, convenient storage, and adjustable catalytic capabilities.^7,8^ Nanozymes are synthetic materials that possess enzyme-mimicking properties and are characterized by their nanoscale structure. The pioneering work on nanozymes was carried out *via* Gao and coworkers in 2007, during which they identified the peroxidase (POD)-like capabilities of Fe_3_O_4_ nanoparticles (NPs).^9^ Since then, nanozymes have been created utilizing a variety of nanomaterials like noble metal NPs, carbon-based nanomaterials, MOFs (metal–organic frameworks), COFs (covalent organic frameworks), and semiconductor metal oxide NPs. However, bulk nanozymes have been limited by their active sites and surface area exposure for enzymatic catalysis, which hinders the reaction performance.

With modern characterization methods like X-ray absorption near-edge spectroscopy and aberration-corrected high-angle annular dark-field scanning transmission electron microscopy, the single-atom catalysts (SACs) were recognized and have generated a growing interest within the domain of heterogeneous catalysis.^10^ SACs featuring atomically dispersed metal sites on their supports optimize the atomic utilization efficiency and enhance the accessibility of a well-defined active site density. Decreasing the size of metal materials to the atomic scale, in conjunction with the incorporation of metalloenzyme-like catalytic centers, imparts exceptional catalytic activity to SACs. For example, Lee and colleagues incorporated the Fe–N_4_ active site, which is a precise copy of the hemin cofactor, into graphene. This modification resulted in a remarkable 700-fold enhancement in catalytic activity, while also demonstrating exceptional selectivity for hydrogen peroxide (H_2_O_2_).^11^ In 2019, a novel subcategory of nanozymes, referred to as single-atom nanozymes (SAzymes), was introduced^12^ and quickly transformed the research frontier in the nanozymes field. SAzymes are distinguished by their optimal atom utilization, high density of active sites, and well-defined electronic and geometric structures, offering clear models for mechanistic investigations.^13,14^ In terms of catalysis, SAzymes are notable for their unique construction that effectively harmonizes and merges the strengths of heterogeneous and homogeneous catalysts.^15^ The active places are distributed almost uniformly across the support substrates, thereby enhancing the selectivity, activity, and stability of the nanoparticles (NPs). Datye *et al.*, for the first time, successfully synthesized SACs by employing cerium dioxide (CeO_2_) to trap Pt species.^16^ In Yan's study, small CeO_2_ clusters were employed as supports, resulting in a preferential orientation of monatomic platinum (Pt) on the (111) crystal plane of CeO_2_.^17^ In comparison to CeO_2_ cluster nanozymes, Pt/CeO_2_ SAzymes demonstrated catalase (CAT)-like function that was approximately 10-fold greater, as well as superoxide dismutase (SOD)-like reactivity that was about four times more potent. Additionally, the polymerase function of Pt/CeO_2_ SAzymes was found to be 3–10 times higher, with the primary reaction rates also increasing by a factor of eight. Moreover, the SAzymes exhibited extended catalytic activity without any significant deterioration over a period of one month, demonstrating consistent performance. It is important to highlight that certain unique SAzymes, like Fe–N_*x*_ SACs, and their coordination environments at single-atom sites can replicate the configuration of natural enzyme active sites. These characteristics render them as ideal candidates for the construction of biosensors that enable the precise analysis of target molecules at very low concentrations.^18^ Therefore, in this study, we provide a comprehensive summary of the approaches used to enhance the enzyme-like features of SAzymes and their catalytic behaviors by examining the various factors that influence these attributes. Furthermore, we discuss recent progresses in the use of SAzyme-derived nanoprobes for the on-site identification of pesticide residues in agricultural food products, with a specific focus on organophosphate pesticides (OPPs), carbamates, acetamiprid, pyrethroids, and other pesticides. We ultimately examined the limitations and challenges associated with scaling up SAzymes for commercial use in food contaminant analysis.

## 2. Catalytic mechanisms and enzyme-like properties of SAzymes

A distinct class of nanozymes, known as SAzymes, exhibits superior catalytic efficiency and selectivity compared to their macroscale counterparts. SAzymes are characterized by the presence of atomically dispersed catalytic sites that exhibit metalloprotease-like properties. Due to their inherent characteristics, SAzymes hold considerable promise for elucidating the catalytic functions of nanozymes, while also serving as a bridge between macroscopic nanozymes and natural enzymes. The active sites of supported SAzymes are situated at the coordination bonds between carriers and metal atoms, which endows them with significant catalytic activity and specificity toward substrates.^19^ However, there remain many controversies surrounding the catalytic mechanism and active sites of SAzymes.^20^ It has been proved that elements like the electronic structure, oxidation state of the metal atom, and surrounding coordination situation impact the catalytic selectivity and activity of SAzymes.^21^ The widely recognized catalytic mechanisms of nanozymes are the electron transfer mechanism, the Fenton-like reactions, and Fenton reaction.^22^ The Fenton reaction involves the generation of ROS (reactive oxygen species), such as hydroxyl radicals (·OH), which possess significant oxidizing capabilities. This process is catalyzed by ferrous ions (Fe^2+^) in the presence of hydrogen peroxide.^23^ In particular, the O–O bond of H_2_O_2_ that is adsorbed onto the SAzymes surface undergoes cleavage, resulting in the formation of two ·OH. Subsequently, ROS oxidize the organic substrate, leading to the generation of alkyl radicals, which in turn elicits a significant colorimetric response. However, Haber and Weiss posited that ·OH should be regarded solely as an intermediate oxidizing agent.^24^ In the context of electron transfer mechanisms, the product and substrate are sequentially bound to or liberated from the enzyme in an alternating manner, a process referred to as the ping–pong mechanism. The process involves two half-reactions: initially, an electron or group is transported from the substrate to the enzyme, leading to a conformational alteration in the enzyme and the discharge of the first product. Following this, the second substrate interacts with the modified enzyme, producing the second product and finally restoring the enzyme to its original state.^25,26^ This procedure operates in a cyclical manner, allowing the reaction to sustain itself. The presence of parallel Lineweaver–Burk plots corresponding to varying substrate concentrations typically suggests that the reaction adheres to the usual ping-pong mechanism.^27^ In view of the wide applications and varied catalytic features of SAzymes, this section outlines the enzyme-like properties of different SAzymes considered over the past decade.

### 2.1. Superoxide dismutase-like SAzymes

The catalytic function of SOD is demonstrated through the process of converting superoxide radicals (·O_2_^−^) into oxygen (O_2_) and hydrogen peroxide (H_2_O_2_) through dismutation.^28^ The SOD-like function of SAzymes is commonly explored using a colorimetric assay that employs a 2-[2-methoxy-4-nitrophenyl]-3-[4-nitrophenyl]-5-[2,4-disulfophenyl]-2*H*-tetrazolium (WST) system or riboflavin and NBT (nitro blue tetrazolium) complex as the reactants. This colorimetric signal typically results in the formation of a chromogenic formazan system, which is then evaluated spectrophotometrically for either the NBT-riboflavin system (560 nm) or WST system (450 nm). Various SAzymes showing SOD-mimicking activity have metals like Mn, Pt, Au, Ni, Cu, Fe, Rh, Mo, and Co.^29,30^ SAzymes exhibiting SOD-like performance can be synthesized through the utilization of various materials like carbon dots, fullerene, MOFs, and graphene quantum dots. In a study, Fe–N/C-CNT SAzymes with Fe–N_3_ units were deliberately arranged and effectively synthesized. The resulting Fe–N/C-CNT SAzymes exhibited tremendous OXD-like catalytic performance. Moreover, a comprehensive study was conducted to analyze the structure-dependent OXD-like activity of the Fe–N/C-CNT SAzymes. The findings revealed that the Fe–N_3_ species played a pivotal role as the active sites mimicking OXD in the Fe–N/C-CNT SAzymes.^31^ In a very recent study, post-synthetic alteration of Cu-BTC (BTC = 1,35-benzene tri carboxylate) MOFs was documented as a method to generate Mn/Cu–C–N_2_ SAzymes.^32^ The SOD-like function of the as-prepared Mn/Cu–C–N_2_ was explored through the utilization of the colorimetric NBT-riboflavin inhibition examine. The SOD-like function of Mn/Cu–C–N_2_ doubled in comparison to the original Cu-BTC MOFs. The enhanced SOD-mimicking function of Mn/Cu–C–N_2_ was ascribed to the following: (i) the carbonization framework is utilized to decrease the activation energy in the NBT inhibition examine; (ii) the boosted SAzymes affinity towards O_2_˙^−^ is attributed to the collective influence of the bimetallic sites.

### 2.2. Peroxidase-like SAzymes

Natural POD can facilitate the oxidation of substrates using H_2_O_2_ as a catalyst. To date, numerous nanozymes with peroxidase-mimicking catalytic functions have been effectively developed since Yan's research team first found the inherent POD-like catalytic performance of the Fe_3_O_4_ NPs.^33,34^ Currently, a significant number of SAzymes utilizing metals, carbon, and zeolites/MOFs have been explored for their exceptional POD-like activity.^35–37^ Compared with natural peroxidase, nanomaterial-based peroxidase mimics are extensively employed in biomedical applications for their low cost, simple preparation, outstanding catalytic stability, high specific surface area, and high stability under harsh sensing conditions.^38^

**Fig. 1:**
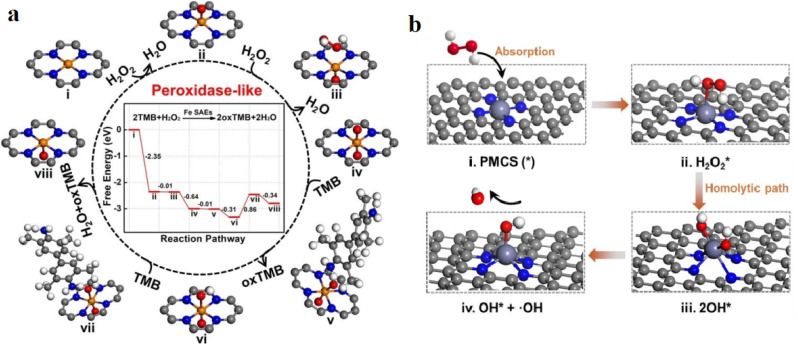
(a) Diagram of the catalytic reaction procedure of the peroxidase route of SAzymes. This figure has been reproduced from ref. 40 with permission from the Royal Society of Chemistry, copyright 2019. (b) Suggested peroxidase-like catalytic performance of PMCS. This figure has been reproduced from ref. 41 with permission from Wiley, copyright 2019.

It is noteworthy that the case studies described consistently utilize H_2_O_2_ and TMB as chromogenic platforms for measuring the activity of POD-mimicking SAzymes. This preference can be attributed to several characteristics of TMB, including its colorimetric response, strong affinity for the POD catalytic unit, and low synthesis cost.^39^ SAzymes with POD-like activity can catalyze the oxidation of colorless *o*-phenylenediamine (OPD) and TMB as substrates, resulting in the production of colored products in the presence of H_2_O_2_. It should be noted that Zhao and colleagues explored the POD-mimicking catalytic function of Fe SAzymes, which were capable of catalyzing the decomposition of hydrogen peroxide to generate free radicals.^40^ Additionally, density functional theory (DFT) assessments were employed to further validate of the POD-like catalytic response procedure of Fe SAzymes. Based on an FeN_4_ site embedded in graphene as an experimental model, the POD-mimicking function of Fe SAzymes was investigated. The energy profile and reaction pathway of the POD-like catalytic activity on the confined FeN_4_ site are illustrated in Fig. 1. Initially, the H_2_O_2_ molecules can spontaneously detach from the FeN_4_ sites, leading to the release of an H_2_O molecule and the creation of a Fe@O intermediate. Afterwards, the formation of an O@Fe@O intermediate led to the dissociation of another H_2_O_2_ molecule at the opposite side of the central FeN_4_ sites. Following this, the presence of extra O species in the O@Fe@O structure proved advantageous for the adsorption of the initial TMB molecule by facilitating the formation of an O–H bond. Consequently, a hydrogen atom from TMB was readily removed to produce O@Fe–OH and oxTMB. Carbon nanomaterial derived from ZIF-8, comprising atomically dispersed Zn atoms, exhibited exceptional POD-like catalytic activity and functioned as a highly effective SAzyme.^41^ The study thoroughly examined the structural development of prepared SAzymes in order to elucidate the relationship between the structure and activity of SAzymes. Furthermore, through the use of DFT evaluations, it was determined that the coordinatively unsaturated Zn–N_4_ building served as the functional site of the SAzymes. The catalytic performance of the SAzyme is illustrated in Fig. 1B. The H_2_O_2_ molecules were taken up by the active sites of Zn–N_4_ within the SAzyme (i), resulting in an adsorption energy of −0.45 eV(ii). The triggered hydrogen peroxide underwent easy dissociation by a homolytic pathway, causing the production of two hydroxyl radicals (iii). Subsequently, a –OH detached from the lone zinc site, leading to the generation of OH* and the active hydroxyl radical (iv).

**Fig. 2:**
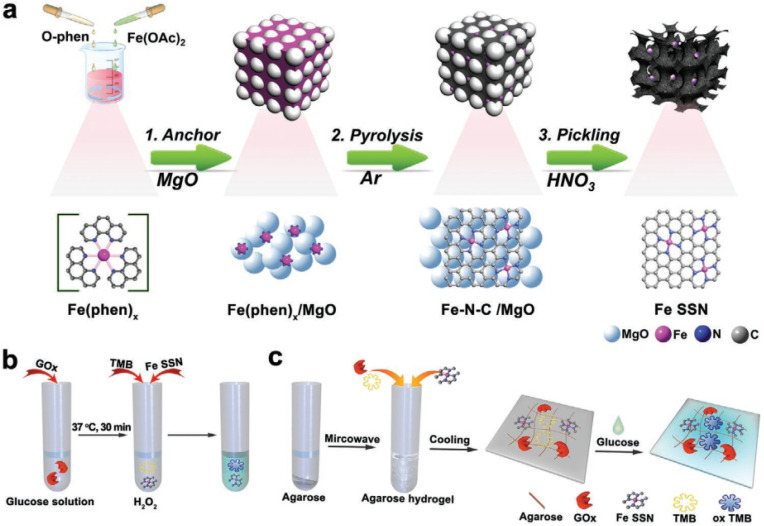
(a) The schematic illustration for the preparation of Fe SSN. (b) Modified colorimetric detection of glucose. (c) Integrated agarose-based hydrogel colorimetric detection of glucose. This figure has been reproduced from ref. 42 with permission from Wiley, copyright 2020.

In the meantime, atomically dispersed single Fe SSN (iron site nanozyme) was also created for the purpose of detecting glucose.^42^ In this study, Fe SSN was produced through the thermal decomposition of a hybrid material consisting of Fe(phen)_*x*_ supported on magnesium oxide (Fig. 2). The as-synthesized Fe SSN complex demonstrated peroxidase-like activity by converting hydrogen peroxide into hydroxyl radicals in the presence of TMB, with an absorbance peak at 652 nm. The *ν*_max_ and *K*_m_ rates for the reaction of Fe SSN with H_2_O_2_ are determined to be 1.32 × 10^−7^ M s^−1^ and 0.36 mM, respectively. The resultant values for Fe SSN with TMB are 2.04 × 10^−7^ M s^−1^ and 0.53 mM, respectively. The *K*_m_ values for H_2_O_2_ were observed to be ten times lower than those found with HRP, indicating the Fe SSN's strong affinity for H_2_O_2_. The Fe SSN was additionally examined for the colorimetric identification of glucose through the utilization of an agarose-based hydrogel. The linear range and limit of detection (LOD) toward glucose by the hydrogel-derived probe were 0.3–10 mM and 0.3 mM, respectively.

**Fig. 3:**
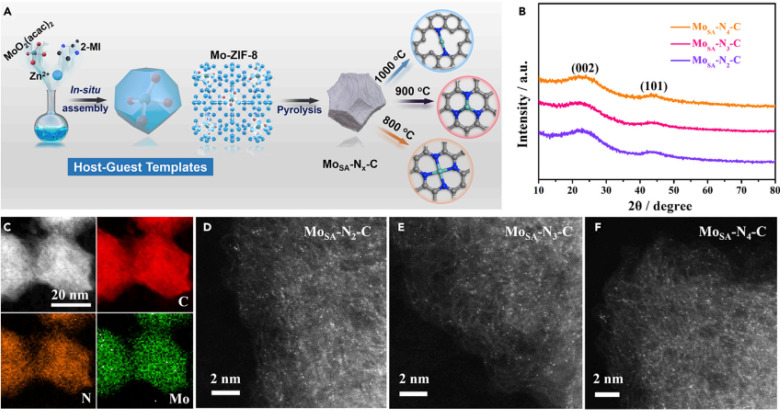
Diagram of the synthesis of Mo_SA_-NA-C and its morphological/crystalline features: (A) schematic of the synthesis method and (B) XRD spectrum, (C) HAADF-STEM elemental mapping and (D–F) HAADF-STEM images. This figure has been reproduced from ref. 43 with permission from Elsevier, copyright 2021.

Currently, both experimental and theoretical investigations were carried out to produce a series of molybdenum single-atom nanosize enzyme (MoSA–N_*x*_–C) by manipulating the coordination amounts of individual Mo places.^43^ The Mo–N_*x*_–C SAzyme series was synthesized through Mo-ZIF-8 MOF pyrolysis deposited on N-doped porous carbon platforms (Fig. 3). The ability of various Mo–N_*x*_–C SAzymes to mimic the POD activity was analytically assessed through the colorimetric oxidation of TMB at 652 nm. Among the catalysts evaluated, Mo–N_3_–C demonstrated the highest peroxidase-like activity. X-ray absorption fine structure (XAFS) spectroscopy was employed to demonstrate that the quantity of N atoms conjugated with Mo played an important role in identifying the POD specificity and activity of the Mo–N_3_–C catalyst. These studies elucidate the relationship between the selectivity and structure, providing a crucial approach for the intentional design of highly specialized nanozymes.

### 2.3. Catalase (CAT)-like SAzymes

The CAT enzyme is found in all cells of aerobic organisms, and plays a crucial role in maintaining cellular ROS levels by catalyzing the breakdown of H_2_O_2_ into O_2_ and H_2_O.^44,45^ CAT has been proposed as a potential therapeutic intervention for various oxidative stress-related conditions, including cancer and aging. Furthermore, CAT has shown significant potential for a wide array of industrial applications, such as food packaging, the dairy industry, wastewater treatment, and other related fields.^46,47^ In view of the antioxidant properties of CAT, numerous researchers have tried to develop synthetic analogs in the metal-centered SAzyme format. The CAT-mimicking function of all SAzymes was utilized to monitor O_2_ evolution, either through the use of a dissolved O_2_ meter or *via* UV spectrophotometric analysis at a wavelength of 240 nm. Ma and colleagues reported on the multifunctional enzyme activity of Fe-NC SAzymes in this context.^30^ They created a Fe–N–C SAzyme by pyrolyzing iron phthalocyanine (FePc) within a zeolitic imidazolate framework-8 (ZIF-8) encapsulation. The cyclic voltammetry study of the Fe–N–C SAzyme in response to the H_2_O_2_ verified the O_2_ evolution along with the H_2_O formation. In the steady-state kinetic analysis of Fe–N–C-facilitated O_2_ production in relation to varying H_2_O_2_ concentrations, it was observed that the decomposition of H_2_O_2_ followed the Michaelis–Menten kinetics model. Additionally, the TOF (turnover frequency) per active site of the Fe–N–C SAzyme was determined to be 1809.34 min^−1^. These authors also demonstrated the antioxidant capabilities of the Fe–N–C SAzyme in HeLa cells that were subjected to b-lapachone (a source of ROS). Currently, PtN_4_C SANzyme has been introduced to remove ROS under tumor microenvironment (TME)-stimulated situations, such as GSH dearth and intracellular H_2_O_2_ build-up.^48^ The SANzyme displayed SOD- and POD-mimicking properties with the conversion of O_2_˙^−^ into H_2_O_2_ and ·OH production, respectively. The SOD-like function of PtN_4_C-SANzyme facilitated the internal generation of H_2_O_2_, which then acted as a precursor for SANzyme to induce the cyclic accumulation of O_2_ and ·OH specifically at the tumor site.

### 2.4. Oxidase-like SAzymes

Natural oxidases have the ability to facilitate the oxidation of substrates, resulting in the generation of oxidized products H_2_O/H_2_O_2_/O_2_˙^−^ in response to the O_2_ presence or other oxidants. Fe–N–C SAzymes with the atomically dispersed metal active sites exhibited exceptional OXD-like catalytic activity, facilitating the conversion of O_2_ into ROS, superoxide radicals 
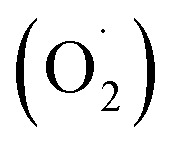
. Furthermore, in the presence of oxygen, Fe–N–C SAzymes can facilitate the oxidation reaction of TMB, leading to the formation of the oxidized state of TMB (oxTMB), which exhibits a blue color.^49^ Likewise, there have been reports of Fe–N–C catalysts containing Fe–N_*x*_ active sites exhibiting OXD-like catalytic activity, enabling the reduction of O_2_ to produce ROS such as 
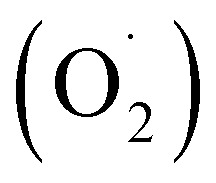
, H_2_O_2_, and ^1^O_2_.^50^ In a study, Fe–N/C-CNT SAzymes featuring Fe–N_3_ units were deliberately developed and effectively synthesized. The designed Fe–N/C-CNT SAzymes revealed outstanding OXD-like catalytic activity. Furthermore, a comprehensive investigation was conducted to analyze the structure-dependent OXD-like activity of the Fe–N/C-CNT SAzymes. The results revealed that the Fe–N_3_ species played a crucial role as the active sites, effectively mimicking the oxidase activity in Fe–N/C-CNT SAzymes. Moreover, it has been discovered that the catalytic process of Fe–N/C-CNT SAzymes, which mimics the OXD catalytic activity, may depend on the involvement of ROS generated during the activation of O_2_.^31^ In another work, the basic procedure of the OXD-like activity of FeN_5_SA/CNF boosted enzyme-like active sites was discovered. To investigate the underlying reasons for the OXD-mimicking catalytic activity of the FeN_5_SA/CNF SAzyme, DFT calculations were performed to analyze the reduction mechanism of the oxygen molecules on the metal single-atom sites. This analysis involved the use of TMB molecules as a reducing agent under acidic conditions. Four catalysts (FeN_4_ SA/CNF, FeN_5_ SA/CNF, MnN_5_ SA/CNF, and CoN_5_ SA/CNF) were investigated due to their OXD-like catalytic activity, as the NiN_5_ SA/CNF and CuN_5_ SA/CNF catalysts exhibited minimal OXD-mimicking catalytic activity and infeasible transition states. The order of the calculated OXD-like activity for the single-atom OXD mimics was determined by evaluating the free energy associated with the oxygen reduction reaction, with FeN_5_ SA/CNF displaying the highest activity, followed by MnN_5_ SA/CNF, CoN_5_ SA/CNF, and FeN_4_ SA/CNF. According to the DFT calculations, the remarkable catalytic activity of SAzyme in mimicking OXD is primarily attributed to the steric configuration of SAzyme and the central metal atoms. Moreover, through theoretical calculations and experimental studies, it was determined that the FeN_5_ SA/CNF catalyst exhibited the most significant OXD-like activity due to the essential synergistic effect and electron donation mechanism when utilizing OXD catalysis as a reference reaction.^12^ In another study, a FeN/C SAzyme characterized by a high level of efficiency and an abundance of iron single atoms coordinated at Fe-NX sites was found to exhibit exceptional OXD-mimicking catalytic activity. The synthesized Fe–N/C SAzyme, which possesses OXD-like activity, can catalyze the oxidation of colorless TMB by dissolved oxygen, resulting in the production of oxTMB.^51^

## 3. Synthetic strategies to fabricate SAzymes

This unique feature affords SAzymes with a multitude of advantages such as dispersed active sites, low cost and variety of synthetic methods over natural enzymes, making them an enticing prospect for various applications in industrial, medical and biological fields (Table 1). So far, the synthetic strategies engaged to design SAzymes comprise the following techniques. Wet chemical technique is a common bottom-up strategy, which normally includes a sequential procedure of primarily enabling metal precursors on the support surface through electrostatic absorption, chemisorption, or co-precipitation. This technique primarily relies on the two fundamental principles of enhancing metal–support interactions and inhibiting the aggregation of atoms. In these strategies, precursor materials that contain metal atoms and single-atom species are chemically dispersed onto the supports. The presence of surface defects on the supports creates anchoring sites for single atoms, thereby preventing their aggregation through strong metal–support interactions. The typical approach for synthesizing single-atom catalysts involves a series of sequential processes, such as drying, calcination, and the reduction and activation of single metal atoms to remove the unreacted ligand. Common wet-chemical synthesis methods, such as the impregnation and co-precipitation techniques, have been utilized in the preparation of SAzymes. The impregnation method, in particular, is a straightforward approach for fabricating SAzymes. This method involves mixing a solution of active metal precursors with a support, allowing the precursors to adhere to the support. To date, the methods for capturing isolated metal atoms on carriers based on the enhanced metal–support interactions mainly include spatial confinement, coordination stabilization, and defect anchoring strategy. The spatial confinement technique comprises attaching metal precursors to the porous materials surface, preventing the migration and aggregation of metal atoms (*e.g.*, MOFs and zeolitic imidazolate frameworks [ZIFs]^52,53^). The spatial confinement technique enables the tailoring of SAzymes with distinct functionalities through the adjustment of the pyrolysis temperature. Atom trapping based on the high-temperature technique is carried out through evaporating the metal atoms, followed by immobilizing them at surface defects of nitrogen-doped support, thereby protecting them from agglomeration and movement. In the strategy for coordination stabilization, bonds between metal and nonmetal are formed through unsaturated coordination atoms on supports and the vacant orbitals of metal atoms, thereby greatly improving the interactions between the metal and the support.^54^ The surface defects of the support provide multiple anchoring sites that capture metal atoms, preventing their aggregation into nanoparticles or nanoclusters.^12^ For instance, Kim *et al.*^11^ synthesized Fe–N-rGO by combining iron precursors with graphene oxide (GO) produced through a modified method. This mixture was subsequently freeze-dried and heat-treated in an ammonia environment. By integrating the Fe–N_4_ unit site into graphene, Fe–N-rGO acquired an active site similar to the natural horseradish peroxidase heme (HPR) cofactor present in the enzyme. Additionally, Cheng's team^55^ developed a novel SAN by anchoring individual Fe atoms onto carbon nanotube (CNT/FeNC)-loaded n-doped carbon. During the wet chemical synthesis, pyrrole molecules were adsorbed onto oxidized carbon nanotubes (CNTs) due to the π–π interactions. Fourier transform extended X-ray absorption fine structure (EXAFS) analysis revealed a distinct Fe–N peak at 1.4 Å in CNT/FeNC, indicating a complete monoatomic dispersion of Fe. Furthermore, techniques like electro-filtration allow for the synthesis of single atom catalysts at room temperature. This method prevents the aggregation of metal atoms, resulting in a uniform distribution of single atoms, which enhances catalytic performance.

**Table 1:** Comparison of the characteristics of SAzymes with those of natural enzymes

Characteristic	Natural enzymes	SAzymes
Stability	Sensitive to changes in harsh conditions (*e.g.*, temperature, pH, and ionic conditions)	More stable under extreme conditions
Reusability	Typically, lower reusability	Can often be reused multiple times in industrial processes, especially with immobilization
Catalytic activity	Specific and efficient in catalyzing biochemical reactions	Boost the catalytic performance by the synergetic collaboration effect from different single atoms, the supports, or doping
Biocompatibility	Biocompatible	Improved-biocompatibility *via* surface engineering of SAzymes
Cost	Less expensive, especially when sourced from biological systems	More expensive to design and synthesize
Applications	Might not be suitable for various applications	Suitable for novel applications, especially in areas like sensory systems, bioengineering, and drug development due to unique electronic/geometrical structural advantages

An alternative synthetic approach (top-down) avoids the high-temperature sintering of nanoparticles (NPs), but it necessitates a specialized strategy to break the robust metal–metal (M–M) bonds in metal precursors, while capturing the released metal atoms using appropriate carriers. The techniques for seizing the liberated metal atoms through metal-support interactions resemble those previously mentioned above.^56^ Direct pyrolysis of the metal precursors that include thermodynamically unstable metal species, such as zinc, mercury, and cadmium, can be effectively utilized to produce SAzymes. Ding *et al.*^57^ synthesized NiS_*X*_ nanoparticles on cadmium sulfide (CdS) NPs to create a volatile CdS–NiS_*X*_ composite, which was then combined with polyacrylonitrile through an electrospinning technique. During the high-temperature pyrolysis process, the sulfur species initially volatilized, establishing carbon frameworks, while the cadmium species rapidly evaporated after reaching 765 °C, resulting in the formation of hollow macroporous fibers. The remaining nickel species were reduced to nickel NPs at 850 °C. Further pyrolysis at 950 °C resulted in the formation of nickel single-atom catalysts. Room-temperature methodologies such as defect engineering and chemical reduction not only require less energy compared to high-temperature pyrolysis, making them more sustainable, but also can achieve better control over the size and distribution of active sites, leading to improved catalytic properties. This broadens their applicability in various fields, including catalysis and biomedical applications.^29,58^

## 4. Main elements influencing the enzymatic characteristics of SAzymes

In contrast to natural enzymes, the catalytic functions of typical nanozymes, which possess inherent enzyme-mimicking characteristics, are primarily influenced by factors such as structural arrangement, dimensions, morphology, and the presence of coordinatively unsaturated dangling bonds.^59,60^ For instance, horseradish peroxidase (HRP) and ZIF-8-encapsulated glucose oxidase (GOx) composites were employed to achieve chromogenic detection of glucose using ABTS. The superior catalytic performance of this system can be attributed to the confined environment provided by ZIF-8, along with its remarkable physicochemical stability.^61^ In the context of SAzymes, the presence of a single atom results in a simplified and distinct atomic arrangement, as well as high intrinsic activity and precise identification of the specific metal center.^62^ As an example, SAzymes, which consist of a single-atom of iron anchored onto a single layer of 2D N-doped graphene (Fe/NG), was employed for the colorimetric detection of Cr(vi) in the presence of 8-hydroxyquinoline as an inhibitor and TMB (a colorimetric sensing probe). The designed SAzymes have a LOD of 3 nM with a linear concentration range of 30 nm-3 μM. The good sensitivity of SAzymes based on Fe/NG can be attributed to its 100% utilization efficiency of Fe atoms and its 2D FeN–C building, which features well-dispersed Fe particles.^63^ In another study, a zinc-based PMCS (porphyrin-like structure) mimicking a POD was established from ZIF-8.^41^ The developed PMCS SAzyme exhibited high catalytic efficiency in oxidizing TMB, which can be attributed to the presence of atomically dispersed single Zn sites. Notably, the coordination number of the single atom is a critical factor in evaluating the catalytic efficiency of SAzymes.^64^ Similarly, a SAzyme using FeN_3_P was developed to inhibit the proliferation of tumor cells.^65^ The SAzyme was synthesized by incorporating Fe ions and monomers of PZM onto the ZIF-8 surface, leading to the development of a Fe/ZIF-8@PZM nanocomposite. The FeN_3_P-based SAzyme was characterized experimentally and theoretically using XAFS analysis, which identified three coordination shells consisting of Fe–C, Fe–P, and Fe–N/O, with coordination numbers of 4.3, 1.1, and 3.6, respectively. The well-organized electronic configuration of the single iron active site, along with its optimal coordination with nitrogen, phosphorus, and carbon atoms, contributes to its exceptional catalytic performance across a wide range of temperatures (30–60 °C) and pH levels (3–5). The measured specific activity of the FeN_3_PSANzyme clearly indicated its superior performance compared to the other nanozymes employed in this research.

The engineering and rational design of the active centers of SAzymes are highly significant and should not be overlooked. Certain SAzymes possess active sites that closely resemble those found in natural enzymes. This design feature has the potential to enhance the catalytic activity and specificity of conventional nanozymes. For example, Lin and coworkers developed a type of Fe–N–C-based SAN, and this SAN possesses a lot of atomically isolated Fe–N_*x*_ active sites.^66,67^ Such configuration could efficiently mimic the active sites of heme enzymes. In this study, the researchers employed the zinc-atom-assisted technique to isolate iron within the polypyrrole-derived carbon nanowire precursor. After polymerization, multiple pyridinic nitrogen atoms are incorporated into the carbon matrix whereas also fixed with Fe, and the Zn ions are evaporated. Finally, its POD-like activity reached 42.8 U mg^−1^. Dong and colleagues also regulate the coordination number of nitrogen, specifically as FeN_5_, which is analogous to the active site of cytochrome P450.^12^ The authors investigated the host-guest architecture of MOF-encapsulated iron phthalocyanine (FePc), where the isolated FeN_4_ sites of FePc undergo reconstruction when subjected to heat treatment. Ultimately, the pyridinic nitrogen species can generate additional FeN_5_ sites, aided by the confinement effect of the MOF. These active sites exhibit favorable oxidase-like activities. The coordination number plays a crucial role in influencing the activity of SACs. This phenomenon is primarily attributed to the diverse nitrogen coordination environments, which can modulate the electronic density of the central metal and, consequently, influence its catalytic performance. Another important factor to consider is the capacity to regulate the coordinated nitrogen with precise numerical values and structural configurations, allowing for an accurate replication of the active site of the natural enzyme. In this regard, a report indicates that SAzymes with FeN_5_ activity exhibited significantly enhanced OD-like capabilities compared to SAzymes centered around FeN_4_, with a performance level ranging from 30 to 1000 times higher than that of conventional nanozymes.^12^ In another study, Cui's team found that the oxygen molecule exhibits a preference for adsorption onto the FeN_3_ site surface in a side-on orientation. Through theoretical analysis, they further determined that the affinity of O_2_ towards FeN_3_ is notably greater compared to FeC_3_, FeN_4_, and FeN_5_.^31^ The metal site coordination environment of SACs can undergo significant alterations through the incorporation of heteroatoms such as B, P, and S, or the introduction of defects in the materials. Therefore, the integration of heteroatom modifications has the potential to significantly enhance the intrinsic activity of active sites. This strategy has proven to be highly effective in amplifying the enzyme-like performance of SACs. For instance, the Fe–N–C SAC utilizes heteroatoms with varying electronegativities and atomic radii to dope the carbon substrate. This doping results in alterations to the charge densities and electron spin of the Fe–N_4_ center. This redistribution enhances the selectivity and catalytic function of the enzyme, and has been widely applied in the energy field. Chen and colleagues developed a configuration in which the Fe–N_4_ species were uniformly dispersed at the atomic level on hierarchical carbon layers decorated with nitrogen and sulfur. This configuration was found to reduce the energy barrier associated with the oxygen reduction reaction (ORR).^68^ The presence of heteroatoms can unevenly affect the redistribution of charges and moderately influence catalytic capabilities. This phenomenon can also be attributed to their impact on the catalytic properties of central metal atoms through long-range interactions. For example, Ding and colleagues engaged chlorine (Cl) to modulate the Fe–N_*x*_ active center through a closely coordinated interaction, resulting in the creation of SAC with uniform FeN_4_Cl active sites.^57^

## 5. Biosensing applications of SAzymes

Nanozymes have been widely utilized in biosensing applications owing to their numerous exceptional characteristics. In recent years, SAzymes have demonstrated improved enzyme-mimicking capabilities, owing to their abundant and atomically dispersed single active metal atoms. This feature has the potential to significantly enhance the performance of biosensors. In this regard, biosensing applications have been highlighted to underscore their essential uses, including direct colorimetric sensing platforms, enzyme cascade systems based on SACs, SAC-based immunosensors, and lateral flow assays. SAzymes have the ability to catalyze common colorimetric substrates like OPD and TMB to generate significant color signals in the presence of H_2_O_2_, and being effectively employed in colorimetric biosensing for the quantification of multiple analytes (such as alkaline phosphatase (ALP), organophosphorus compounds, ascorbic acid (AA), acetylcholinesterase (AChE) activity, and H_2_O_2_). By using Fe–N–C SAC-based colorimetric biosensing, Lin and colleagues have created a range of paper-based bioassays designed for the precise detection of AA, glucose, and H_2_O_2_.^55^ Soon after, their research team developed a highly sensitive colorimetric biosensing assay for measuring butyrylcholinesterase (BChE) activity by utilizing the Fe–N–C SAC as a replacement for natural HRP. In this biosensing assay, BChE was employed to facilitate the hydrolysis of butyrylcholine (BCh) into thiocholine (TCh). The resulting TCh, possessing specific reducibility properties, was capable of impeding the TMB chromogenic reaction catalyzed by the Fe–N–C SAzyme. With this biosensing technique, BChE activity was identified ranging from 0.1 to 10 U L^−1^, and with a very low LOD of 0.054 U L^−1^.^37^ Currently, colorimetric biosensors that utilize SAzymes have been primarily employed for the detection of various analytes. However, there is a pressing need to advance these biosensors by integrating immunoassay techniques, which would facilitate the specific and sensitive identification of a broader range of analytes. In this context, a new type of Fe–N_*x*_ SAC was developed by modifying Fe-doped polypyrrole (PPy) nanotubes. These Fe–N_*x*_ SAC were subsequently employed as a replacement for HRP in a conventional ELISA kit, demonstrating a heightened sensitivity in the detection of amyloid beta 1–40 66.^69^ The findings suggest that Fe–N_*x*_ SAC achieves a significant concentration of individual active sites at the atomic level. Furthermore, these SAzymes exhibited enzymatic characteristics similar to HRP. This study proves that SAzymes can serve as an effective replacement for enzymes in labeling for ultrasensitive colorimetric immunoassays. Drawing inspiration from natural enzymes, there is potential to enhance the performance of enzyme-mimicking catalysts by strategically designing the coordination structure of the active site at the atomic level. Wu and colleagues developed an axial-ligand-modified Fe–N–C SAC that was integrated into a sandwich-type immunoassay for the detection of carcinoembryonic antigen (CEA), referred to as the SAzyme-linked immunoassay.^70^ While ELISA provides increased sensitivity and throughput in comparison to traditional laboratory detection techniques, its extensive time requirements render it unsuitable for point-of-care testing (POCT) scenarios. In contrast with ELISA, LFIA represents a highly sensitive, rapid, facile, and cost-efficient technique for detecting targets. SAzymes with excellent stability and enhanced enzyme-like catalytic activity could be a perfect candidate for LFIA to increase their detection capability. For instance, Cai and coworkers developed a very widespread approach to produce Fe–N–C SAC, and applied it in lateral flow immunoassays for the determination of mycotoxins. The method involved the utilization of pyrolysis on iron ions derived from hemin, which were doped into ZIF-8, and the optimized Fe–N–C SAC with the highest effective catalytic function was subsequently selected.^71^ By leveraging the catalytic amplification system, both quantitative and qualitative detection can be readily achieved through visual inspection with the naked eye, or by utilizing a smartphone to observe the colorimetric reaction of the test lines. In another study, a high-performance POD-mimicking SAzyme was engineered and integrated into the corresponding LFIA, resulting in a significant enhancement of the sensing capabilities of the LFIA.^72^ This Fe-SAC was synthesized by utilizing heme-doped ZIF-8 as a precursor, enabling the development of an active site that closely mimics the coordination environment of the active iron center found in the natural enzyme and its associated functionality. Consequently, Fe-SAC achieves atomically distributed Fe active sites on the catalyst interface. This design allows the SAzyme to exhibit enhanced peroxidase-like activity, indicating that Fe-SAzyme has the potential to serve as a highly effective substitute for natural enzymes. The synthesized Fe-SAC serves as a marker in the development of a LFIA (Fe-SAC-LFIA) for the analysis of 2,4-dichlorophenoxyacetic acid (2,4-D). This method explored the exceptional detection capabilities, characterized by superior selectivity and specificity. Significantly, the development of a POC detection platform has successfully achieved a low LOD of 0.82 ng mL^−1^, along with a linear detection range spanning from 1 to 250 ng mL^−1^. This capability has been validated through testing on human urine samples, demonstrating the feasibility of utilizing this platform for assessing a person's 2,4-D exposure level.

In addition to optical biosensing, electrochemical biosensors have garnered significant attention as a suitable platform for integrating SAzymes due to their advantages, including high sensitivity, cost-effectiveness, ease of operation, and the capability for *in situ* real-time monitoring. Furthermore, the combination of SAzymes with electrochemical biosensors has the potential to amplify the electrochemical signal. Zhu's team prepared an atomically dispersed Ir–N–C SAC by using a template-assisted technique, which exhibited brilliant ORR activity.^73^ This Ir SAzyme was coupled with AChE to act as an electrochemical detecting platform for the determination of organophosphorus pesticides. The catalytic current density presented a strong correlation with the logarithm of organophosphorus pesticide concentrations, demonstrating high selectivity and sensitivity. The same group also created a POD-like Fe_3_C@C/Fe–N–C SAzyme as a H_2_O_2_ electrochemical sensor with exceptional sensitivity (LOD = 0.26 μM) and selectivity.^74^ Another electrochemical detection substrate was developed using a single-atom Ru biomimetic SAzyme. Leveraging its high stability, selectivity, and sensitivity in identifying the oxidation of uric acid (UA) and dopamine (DA), this Ru SAzyme-based biosensor can effectively diagnose UA and DA in real biological serum samples. Similar simultaneous UA determination was also realized *via* a Co SAC-derived electrochemical biomimetic probe. The low detection limits and broad linear detection ranges of this biosensor using Co SAzyme could meet the requirements of practical diagnosis. These electrochemical biosensors utilizing SAzyme offer a novel approach for real-time and *in vivo* analysis of living organisms. Mao and colleagues introduced a biosensing platform utilizing a Cu–N_2_ SAzyme for the specific electrochemical H_2_O_2_ reduction reaction (HPRR) rather than ORR.^75^ By virtue of the preference to H_2_O_2_ over O_2_ molecules, a Cu–SAC-based microsensor demonstrated a strong response to H_2_O_2_ without O_2_ interferences. This outcome confirmed the potential practical use of SAzymes for real-time quantitative analysis.

## 6. Application of SAzymes for the determination of pesticide residues

In contrast to traditional synthetic or natural catalysts, SAzymes offer nearly complete atom utilization and distinct atomic dispersion. These characteristics enable SAzymes to demonstrate exceptional efficacy in detecting a wide range of contaminants present in food products. Consequently, this section provides a brief overview of recent literature regarding the application of SAzymes for the determination of various pesticide residues.

### 6.1. Carbamates

Carbamates are one of the widely utilized synthetic pesticides which comprise a variety of herbicides, insecticides, fungicides, molluscicides and nematocides.^76–78^ Diethofencarb and methiocarb are frequently exploited carbamate pesticides known for their fungicidal and insecticidal properties, respectively.^56^ The widespread use of carbamate pesticides in agriculture has raised global concerns due to their potential harmful effects on human health and the environment. Carbamates are compounds that are either aliphatic or cyclic derivatives of carbamic acid. They are readily absorbed through the gastrointestinal tract, lungs, and skin, and have the ability to penetrate the blood–brain barrier, leading to overstimulation of the parasympathetic nervous system.^79^ Nowadays, the increased use of methiocarb as a substitute for organochlorine and organophosphorus pesticides has sparked significant concerns because of its endocrine-disrupting features. Specifically, it exhibits androgen antagonist and estrogen agonist effects, which can have detrimental implications for both human health and the environment over an extended period. Thus, it is essential to employ rapid, accurate, and sensitive techniques for detecting carbamates in order to ensure food safety and mitigate potential threats to human health.

**Fig. 4:**
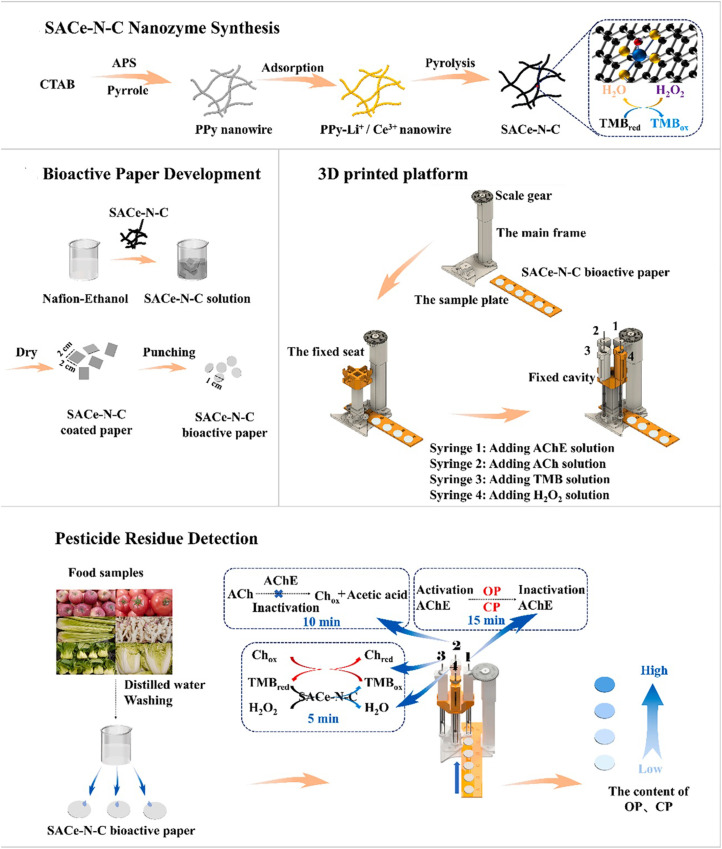
Fabrication mechanism of SACe-N-C nanozyme bioactive paper for sensing organophosphorus carbamate pesticide residues (PPy nanowire: poly-pyrrole; CTAB: cetyltrimethyl ammonium bromide; APS: ammonium peroxydisulfate; OP: organophosphorus pesticides; CP: carbamate pesticides; AChE: acetyl-cholinesterase; ACh: acetylcholine; Ch: choline). This figure has been reproduced from ref. 81 with permission from Elsevier, copyright 2022.

Taking advantage of the exceptional catalytic characteristics of metal–metal bonds, atomic clusters have the potential to enhance the catalytic performance of SACs through the synergistic effects of dual atomic-scale sites. In this context, Co_3_N clusters were employed to facilitate the synthesis of Co SACs using a simple doping technique.^80^ The use of X-ray absorption spectroscopy confirmed the existence of functional metal places in the synergetic dual-site atomic catalysts of Co_3_N@Co SACs as Co_3_–N and Co–O_4_ moieties. Co_3_N@Co SACs proved to be an excellent co-reactant, significantly increasing the chemiluminescence signal by 2155.0-fold, a notably superior effect compared to the 98.4-fold increase observed with pure Co SACs. The dual-site atomic catalysts demonstrated synergistic effects in promoting the decomposition of H_2_O_2_ into singlet oxygen and superoxide radical anions, resulting in exceptional catalytic performance. To assess the feasibility of these catalysts, the potential application of Co_3_N@Co SACs as chemiluminescent nanoprobes was investigated in the development of a highly sensitive immunochromatographic assay for quantifying pesticide residues. In another study, Song's research team developed and validated a SACe-N-C nanozyme as POD-like with exceptional activity.^81^ Based on this work, a paper-derived biosensor was developed by integrating bioactive paper with a 3D printed substrate to facilitate the colorimetric detection of pesticide residues. As illustrated in Fig. 4, the process of sensor construction included the use of Ach, AChE, H_2_O_2_, and TMB onto the bioactive paper to establish a cascade catalytic response method. The newly designed portable biosensor exhibited quick colorimetric sensing (within 30 minutes) of pesticides, such as methamidophos, omethoate, carbosulfan, and carbofuran in various vegetables and fruits samples with LODs as low as 71.51, 55.83, 74.98, and 81.81 ng mL^−1^, respectively.

Tai and colleagues designed 3D porous nanoribbons of graphene oxide doped with cerium (Ce-GONRs) by cross-linking GONRs with Ce^3+^ ions.^82^ Ce-GONRs exhibit outstanding dual enzyme-like capabilities (OXD-like and POD-like), owing to their porous structure and synergistic effects, and they can be reused at least 5 times after recycling. The found that carbaryl (a carbamate insecticide) and diafenthiuron (a thiourea insecticide) were able to significantly impede the dual enzyme-like functions of Ce-GONRs, owing to the multi-interface synergistic influence of hydrogen bonding and π–π stacking. Inspired by this mechanism, the enzyme-free colorimetric probe was developed for the analysis of carbaryl and diafenthiuron. The linear concentration ranges of carbaryl and diafenthiuron were 2–800 ng mL^−1^ and 10–1500 ng mL^−1^, and the detection limit of these were 0.23 and 0.57 ng mL^−1^ in actual lake water and apple samples, respectively.

More recently, a multifunctional SAzymes, referred to as 3D Ni, N-codoped porous carbon (Ni-NPC), was developed to demonstrate exceptional adsorption properties and a range of enzyme-like functions, including peroxidase and oxidase activities.^83^ These characteristics arise from the well-dispersed Ni sites and unique mesoporous thin-shell building. The well-organized adsorption capability of Ni-NPC was evaluated for three carbamate pesticides like carbaryl, isoprocarb, and metolcarb. Moreover, a colorimetric sensing technique for detecting carbamate was designed based on its strong peroxidase-like catalytic function and sequential catalytic interactions with acetylcholinesterase. Additionally, a mobile colorimetric probe utilizing a hydrogel sphere in conjunction with a smartphone platform was developed. This probe allows for the quantitative, on-site, and rapid monitoring of carbamate pesticides, with an impressively low LOD of 1.5 ng mL^−1^. Significantly, this sensor was effectively exploited for assessing carbamate levels in vegetable samples (specifically rape and pakchoi) and lake water, thereby advancing the progress of real-time sensing technologies for environmental and food screening. In another study, Wang's group synthesized POD-like SAzymes with a substantial iridium (Ir) loading of 5.31% on GO nanosheets [Ir(iii)/GO] by a coordination response involving the oxygen-containing groups in GO and the Ir(iii) complex.^84^ The construction approach avoids the use of pyrolysis and nitrogen doping techniques, which are commonly employed methods to enhance the enzyme mimic activity of graphene oxide. Ir(iii)/GO SAzymes exhibit exceptional peroxidase-like activity due to the highly reactive Ir atoms, while lacking oxidase-like activity. Due to the outstanding performance of the POD-like function, a facile and highly responsive colorimetric platform for detecting pesticides has been developed. The fabricated detecting substrate provides a highly effective method for identifying pirimicarb with a “switch-on” mechanism, within a linear range of 10–300 nM and a LOD of 2.81 nM. Furthermore, the recognition system was constructed as a portable testing kit, consisting of a sample processing tube and a testing swab. With the assistance of a color-reading application, the testing kit is capable of sensing pirimicarb at a LOD of 3.31 nM. It is remarkable to achieve such high quantification sensitivity through the utilization of a facile colorimetric approach. This work not only introduces a new technique for synthesizing Ir-based SAzymes, but also reveals the exceptional potential of Ir(iii)/GO in the biosensing field.

**Fig. 5:**
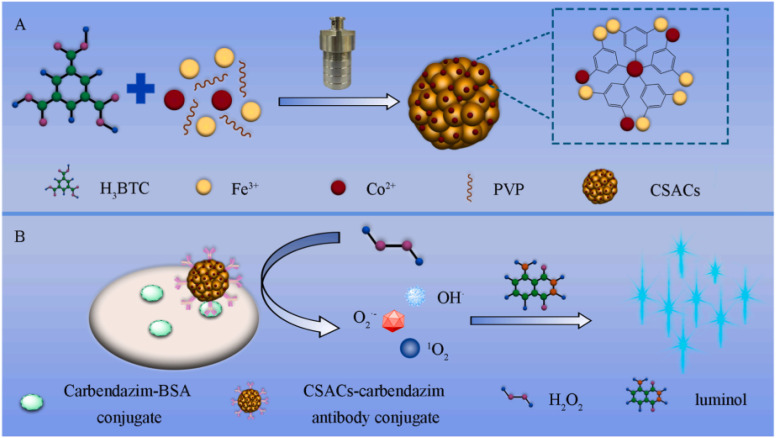
(A) Solvothermal technique employed to synthesize CSACs immobilized on hybridized MOFs Fe_2_O_3_/MIL-100(Fe). (B) Chemiluminescent immunoassay technique used for the detection of carbendazim, with CSACs serving as signal assays. This figure has been reproduced from ref. 86 with permission from Elsevier, copyright 2022.

Owing to their great surface energy, single-dispersed atoms have a tendency to aggregate in the absence of any force acting between the atoms and the supports.^85^ Hence, the selection of suitable carriers for immobilizing single-dispersed atoms, like carbon and metal oxides materials, is crucial for the creation of stable SACs. The process of high-temperature calcination is often utilized to immobilize single-dispersed atoms onto supports and prevent their aggregation. However, this method inevitably leads to poor water dispersibility of SACs and the loss of labeling elements. In this context, Luo and colleagues synthesized hybridized MOFs Fe_2_O_3_/MIL-100(Fe) to serve as carriers for immobilizing cobalt CSACs (Fig. 5).^86^ In addition to the inherent advantages of MOFs, such as good water dispersibility and remarkable porosity, the carriers exhibit significantly enhanced capacity for loading cobalt atoms due to the incorporation of metal oxides Fe_2_O_3_. In contrast to the original MOFs MIL-100(Fe), the carriers prove enhanced loading capability, with the cobalt element being loaded at a level of 4.69 wt%. The introduction of cobalt atoms into hybridized MOFs Fe_2_O_3_/MIL-100(Fe) significantly enhanced the specific surface area of the carriers by 68 times. CSACs at a concentration of 1.0 μg mL^−1^ exhibited the capability to facilitate the conversion of different reactive oxygen species derived from H_2_O_2_, leading to a significant increase in the chemiluminescent emission of the luminol-H_2_O_2_ system by up to 2297 times. Taking advantage of these appealing characteristics, the chemiluminescent immunoassay method applied CSACs as highly responsive signal nanoprobes for identifying carbendazim in Chinese medicinal herbs with a LOD down to 1.8 pg mL^−1^ and a linear range of 10 pg mL^−1^-50 ng mL^−1^.

### 6.2. Acetamiprid

Acetamiprid, a type of neonicotinoid insecticide, has been extensively employed in agricultural and food production for pest management purposes.^87,88^ It has been preferred over organophosphorus pesticides and other conventional insecticides due to its high insecticidal activity and comparatively lower toxicity to mammals. However, the widespread use of acetamiprid has led to adverse effects on non-target organisms, including soil fauna, fish, and bees.^89^ Additionally, residues of acetamiprid have been identified in a variety of food crops, water sources, and soil samples. Prolonged exposure or unintentional ingestion of acetamiprid could pose potential risks to human health.^90^

The majority of the current SAzymes exhibit peroxidase-like characteristics, facilitating the production of various ROS for enhanced bioassays and effective biotherapy applications.^86,91^ Considerable efforts have been dedicated to augmenting their function by increasing the loading quantities of active sites or modifying their coordination environments.^92^ Nevertheless, SAzymes with SOD-like properties have received minimal interest due to their limited utility, except in cytoprotection. It is imperative to address this deficiency by developing SAzymes with antioxidant enzyme-like properties to remove ROS, and expand their applications in biomedicine and bioassays. Manganese oxide (Mn_3_O_4_) has been shown to possess favorable antioxidant properties in the removal of H_2_O_2_ and OH·, thereby prompting investigations into the development of Mn SAzymes with significantly improved efficacy. Herein, a coprecipitation technique conducted at room temperature is proposed for the synthesis of Mn SAzymes incorporated within the structures of PBAs on TMSs, facilitated by the use of large surfactants. This method exhibits excellent water dispersibility and a high loading capacity of up to 13.5 wt%.^93^ Atomically dispersed Mn elements exhibit excellent SOD-like activity in removing O_2_˙^−^, while TMSs have the capability to absorb luminescent signals across a broad spectrum of wavelengths. Therefore, the developed Mn SAzymes displays synergistic quenching ability to the emission of a ROS-based chemiluminescent probe. A competitive immunoassay technique was established for the quantification of acetamiprid on a LFTS substrate, exploiting it as a powerful chemiluminescence quencher. The technique for sensing acetamiprid displays a concentration range of 1.0–1.0 × 10^3^ pg mL^−1^ and a LOD of 0.3 pg mL^−1^. Its accuracy has been confirmed by identifying acetamiprid in medicinal herbs with satisfactory recoveries.

Among SAzymes, Fe–N–C SAzymes are distinguished for their distinctive building, which contains iron atoms bound to carbon and nitrogen atoms in a carbonaceous structure. Fe–N–C SAzymes demonstrate significant catalytic efficiency and selectivity in ORR (oxygen reduction reactions), allowing them to stably and efficiently carry out catalytic processes.^94^ Particularly, Fe–N–C SAzymes are widely utilized as peroxidases for the catalysis of H_2_O_2_ decomposition, leading to the generation of ROS-like superoxide free radicals (O_2_˙^−^) and hydroxyl radicals (·OH). For instance, Feng and colleagues developed Fe–N–C SAzymes that facilitate the conversion of H_2_O_2_ into ·OH, serving as an antimicrobial agent.^95^ Likewise, Wang's group reported on Fe–N–C SAzymes with peroxidase-like properties to detect acetylcholinesterase in the presence of H_2_O_2_.^96^ Hence, the Fe–N–C nanomaterial exhibits unique physicochemical properties, rendering it a promising candidate in the development of aptasensors due to its cost-effectiveness and enhanced stability. A current work initially integrated Fe–N–C SAzymes with a DNA aptamer to create a aptasensor using the Fe–N–C SAzyme to detect cancer cells.^97^ In this platform, DNA aptamers were linked with Fe–N–C SAzymes. DNA aptamers identify the target molecule, while Fe–N–C SAzymes act as a peroxidase to catalyze H_2_O_2_ into OH·, which further oxidizes TMB to oxTMB. This process can be sensed through a colorimetric probe. Nevertheless, the peroxidase function of Fe–N–C SAzymes necessitates the addition of H_2_O_2_, which is destructive and unstable. The existence of H_2_O_2_ restrictions the utilization of the Fe–N–C SAzyme-derived aptasensors. Based on these criteria, Yu *et al.*^98^ introduced a Fe–N–C SAzyme-derived aptasensor to detect acetamiprid in water samples. In this study, a straightforward dual-confinement method was utilized to produce Fe–N–C SAzymes, which were then evaluated for their efficacy as oxidase-like nanozymes. The synthesized Fe–N–C SAzymes offered unique oxidase-like properties, and showed the ability to interact with a chromogenic indicator, TMB, acting as the signal transduction component in their assay. Moreover, they found that aptamers modified with thiol groups were able to efficiently reduce the oxidase-like function of Fe–N–C SAzymes. Through the utilization of the FeN–C-TMB system and Ace-specific aptamers, a novel aptasensor was created, demonstrating remarkable sensitivity (with a LOD of 16.9 nM) and specificity. It also provided favorable recoveries ranging from 99.7% to 101.17% in sensing actual river water samples. This study presents a pragmatic approach for detecting environmental pollution using oxidase-like Fe–N–C SAzymes within an aptasensor.

**Fig. 6:**
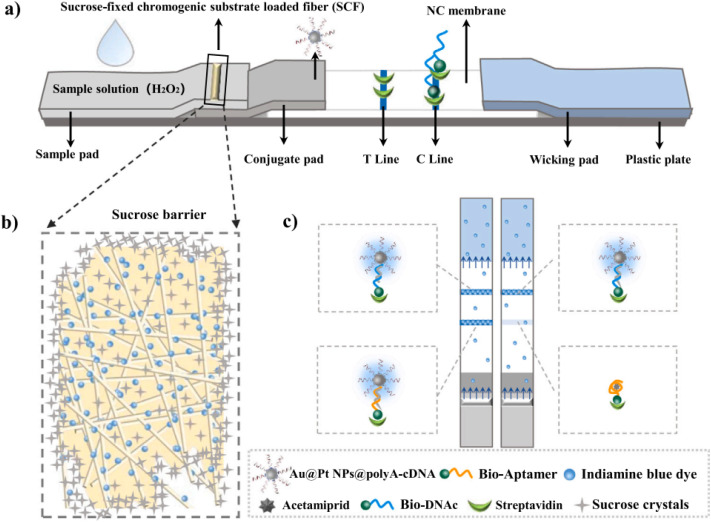
Diagram of the sensing principle of the Apt-CSNLFAs. (a) Configuration of LFAs; (b) representation of the local amplification of RCF; (c) differentiation between positive and negative tests for acetamiprid. This figure has been reproduced from ref. 103 with permission from Elsevier, copyright 2023.

Nanozymes with exceptional catalytic characteristics have emerged as promising signal markers, attracting significant interest among researchers because of their integration with lateral flow assays (LFAs).^71,99^ Most recently, Mao's team introduced a signal-enhanced nanozyme lateral flow assay (NLFA) for detecting acetamiprid in tomato samples based on a bivalent triple helix aptamer.^100^ However, in contrast to conventional LFAs, the NLFAs needed additional operation stages, such as timing the nanozyme capture, dropping substrate, and chromogenic substrate preparation. These necessities clearly increase the error sources and decrease the convenience of operation. In order to address the aforementioned issue, some researchers have integrated reagents to enhance the signal onto LFA strips, allowing for their automated delivery to the test line (T line) of the LFA strip.^101,102^ In this context, more recently, Mao and his team developed an Apt-CSNLFA system that incorporates a chromogenic substrate and nanozyme amplification within LFA strips (Fig. 6).^103^ This innovation allows for the automated and delayed chromogenic substrates release during testing. The precise administration of chromogenic substrates was achieved through the incorporation of a SCF, which was constructed at the end of the sample pad. The substrates that are conveyed automatically have the potential to undergo catalysis for color change through the nanozyme immobilized on the T line. The suggested Apt-CSNLFA has the capability to eliminate the standard procedures associated with NLFA strips, such as timing the nanozyme capture, adding substrate drops, substrate preparation and making it as simple as the conventional Apt-LFAs. This methodology was utilized in an acetamiprid aptamer LFA, and the established Apt-CSNLFA realized a LOD of 0.17 ng mL^−1^ with a concentration range of 1–150 ng mL^−1^ toward acetamiprid. The Apt-CSNLFA exhibited comparable analytical capabilities to traditional aptamer NLFAs, while simplifying the process by eliminating the need for additional steps associated with catalytic signal amplification. Furthermore, the novel approach was utilized for the identification of tomato samples contaminated with acetamiprid. Recovery rates between 95% and 106.4% were achieved, indicating the significant potential for the rapid quantification of acetamiprid.

### 6.3. Organophosphorus pesticides

**Fig. 7:**
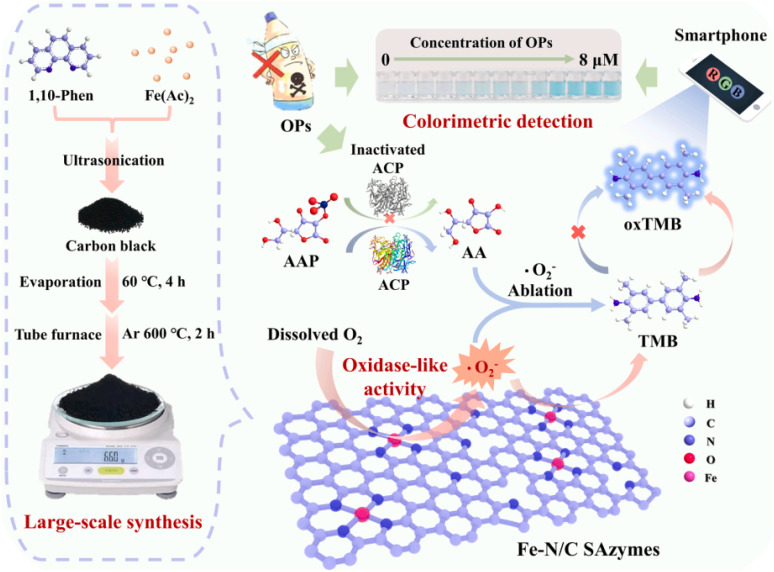
Diagram illustrating the large-scale production of Fe–N/C SAzymes and the colorimetric system utilizing Fe–N/C SAzymes for the determination of OPs. This figure has been reproduced from ref. 114 with permission from Elsevier, copyright 2024.

Organophosphorus pesticides (OPs) represent a predominant category of pesticides used worldwide.^104,105^ Representing at least 20% of the total global pesticide usage annually, these highly efficient and cost-effective pesticides play a crucial role in safeguarding global food security.^106,107^ OPs are synthesized through the reaction of an alcohol with phosphoric acid, where a phosphorus (P) atom prominently participates in forming a double bond with either sulfur or oxygen.^108^ By incorporating different functional groups into organophosphorus compounds, a range of chemicals such as chlorpyrifos, parathion, malathion, methamidophos, and fenamiphos can be synthesized.^109^ At present, OPs have become integral in crop protection. Nevertheless, the excessive application of pesticides has resulted in their accumulation in various environmental media, such as water, soil, and air. This poses a challenge for individuals to avoid contact with environments and food contaminated with OP compounds through skin contact and consumption. According to data from the WHO, around 300 000 people are at risk of life-threatening conditions each year as a result of OP exposure. To safeguard the environment and mitigate further harm, it is pivotal to progress sensitive and accurate biosensors for measuring OP pollutants. Particularly, optical biosensors utilizing fluorescence or UV-vis, in conjunction with portable devices like smartphones and RGB techniques, have garnered significant interest for real-time acquisition and *in situ* signal variations analysis.^110^ The SAzyme-derived optical nanoprobes were reported for the identification of Ops in recent years. In this regard, based on the salt-template approach, the peroxidase-like SAzymes with abundant Cu sites on carbon nanosheets (Cu–N–C) were produced.^111^ The Cu–N–C SAzymes with the highly dispersed active Cu atoms (approximately 5.1 wt%), demonstrate exceptional catalytic activity for mimicking natural peroxidase. A three-enzyme-based cascade response composite was developed by conjugating Cu–N–C SAzymes with choline oxidase and natural acetylcholinesterase. This system was designed for the colorimetric determination of acetylcholine and organophosphorus pesticides. This study not only presents a methodology for the synthesis of SAzymes featuring numerous active sites, but also offers novel perspectives for the development of strong nanozyme biosensing platforms. While SAzymes have the potential to address the limitations of traditional nanozymes, their practical application is often hindered by low metal loading (∼1 wt%) and low yield.^112^ In light of the demands of real-world biosensing applications and facilitating the commercialization of biosensors, it is imperative to first attain the large-scale and highly efficient production of SAzymes.^113^ Hereinto, a large-scale fabrication of Fe–N/C SAzymes is fulfilled through the “ligand-mediated” approach.^114^ In this study, 1,10-phenanthroline is chosen as the ligand to interact with metal cations, and the resultant metal complexes are immobilized on commercially available carbon black prior to undergoing pyrolysis at 600 °C in an argon atmosphere. Fe–N/C SAzymes with a substantial Fe content of 3.9 wt% can be produced on a large scale because of the lower pyrolysis temperature, which reduces metal loading losses and yield losses. The acquired Fe–N/C SAzymes demonstrate efficient and stable oxidase-like catalytic performance, capable of producing O_2_˙^−^ through the direct absorption of dissolved O_2_ within an acidic environment. Consequently, Fe–N/C SAzymes show the ability to facilitate the oxidation process of TMB without the unstable and highly toxic H_2_O_2_, causing an obvious change in hue. Regarding this, a colorimetric biosensor for OPs using Fe–N/C SAzymes was designed by Zhang *et al.* (Fig. 7).^114^ ACP (acid phosphatase) was employed to catalyze the substrate AAP (l-ascorbic acid-2-phosphate) into AA (ascorbic acid), leading to a reduction in the oxTMB production.

The presentation of OPs can prevent ACP activity, causing a decline in the AA production and promoting the color variation of TMB. Nevertheless, AA was generated by the active ACP in the absence of OPs, subsequently decreasing the formation of blue oxTMB. Through the integration of smartphones and RGB analysis, color variation data is converted into digital format to enable *in situ* and real-time measurement of OPs. In this way, the suggested approach can realize accurate and super-sensitive analysis of OPs with a broad concentration range of 1–100 nM and low LOD of 0.4177 nM in actual food samples and environment. Moreover, thanks to the outstanding catalytic function, large-scale synthetic method, and low-cost of the developed Fe–N/C SAzymes, a biosensor using Fe–N/C SAzymes has noticeable marketability benefits.

**Fig. 8:**
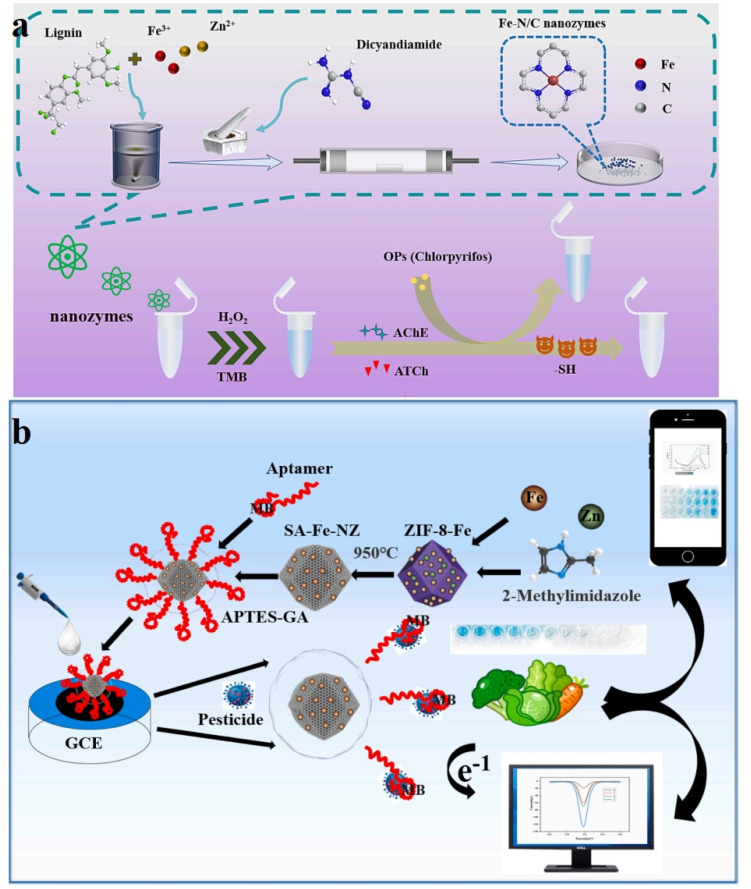
(a) Diagram illustrating the construction procedure of the Fe–N/C SAzymes and the colorimetric sensing approach of OPs. This figure has been reproduced from ref. 116 with permission from Elsevier, copyright 2023; (b) construction process of the colorimetry/electrochemical dual-mode aptasensor based on the Fe-SAzymes quantitative and qualitative identification of multiple OPs. This figure has been reproduced from ref. 117 with permission from Elsevier, copyright 2024.

Based on the Fe–N/C SAzymes, a novel colorimetric smartphone-based system has been developed for malathion detection.^115^ The SAzymes consist of Fe–N_*x*_ groups that are individually dispersed at the atomic level and supported by porous carbon materials derived from MOFs. The as-prepared single-atom nanozymes possessed oxidase-like properties, enabling the conversion of TMB into oxTMB without the need for H_2_O_2_. Additionally, the AA2P, an ACP platform, has the potential to undergo hydrolysis, resulting in the production of AA. The generated AA can decrease the production of oxTMB, leading to a noticeable reduction in the intensity of the blue color. The existence of malathion inhibited the activity of ACP and restricted the production of AA, ultimately facilitating the restoration of the catalytic TMB chromogenic reaction. A new colorimetric assay has been successfully developed for the quantification of malathion with a LOD of 0.42 nM under optimized operational parameters. Furthermore, the method was effectively utilized for quantifying malathion in environmental and food samples using the described approach. Additionally, the effectively integrated paper/smartphone probe enabled the rapid, and sensitive, reliable determination of malathion with a LOD of 1 nM. In another study in 2023, a colorimetric recognizing system was developed to identify OPs by coupling nanozymes with natural enzymes (Fig. 8a).^116^ Fe–N/C SAzymes were synthesized through a one-pot synthesis technique by pyrolyzing alkaline lignin. A variety of characterization techniques were employed to clarify the microstructural features and reaction mechanisms of the nanozymes. In the material, N mostly interacts with Fe in the pyrrole nitrogen form, catalytic hydrogen peroxide will produce hydroxyl radicals (·OH). The Fe–N/C exhibits favorable peroxidase-like characteristics and generates signals in UV detection, following the addition of TMB. Thus, the integration of the catalytic characteristics of Fe–N/C with the incorporation of AChE for the quantification of OPs in soil, exemplified by chlorpyrifos, is proposed. The production of ATCh catalyzed by AChE may impede the color signal. Conversely, the existence of chlorpyrifos hinders the function of AChE and returns the response absorbance. The chlorpyrifos content can be determined by monitoring the alterations in color. The detection ranges for chlorpyrifos using this method was determined to be between 0.05 and 10.0 μg mL^−1^, with a LOD of 2.11 ng mL^−1^. Very recently, Ren and colleagues synthesized two iron single-atom anchored N-doped carbon materials with varying N-coordination numbers through by manipulating of the pyrolysis temperature.^118^ The Fe/CN-800-A catalyst demonstrated remarkable oxidase-like activity and specificity due to the presence of FeN_2_ sites, achieved through pyrolysis at 800 °C, followed by acid leaching. This observation is strongly correlated with the N-coordination number of iron sites, as evidenced by both DFT calculations and experimental analyses. The Fe/CN-800-A catalyst exhibits oxidase-like properties by converting colorless TMB into blue oxTMB. The presence of TCh, a product of AChE-mediated hydrolysis of ACTh, can inhibit this colorimetric reaction, allowing for the determination of chlorpyrifos concentration.

The widespread application of SAzymes is hindered by the irreversible aggregation of single atoms and a limited number of functional groups on their surface. This issue arises from the highly carbonized substrate surface and the relatively harsh conditions of the pyrolysis pretreatment.^119^ Hence, it is crucial to carefully choose a suitable technique for preparing SAzymes and a method for immobilizing bioreceptors. ZIF-8, a significant constituent of MOFs, is employed to uniformly disperse single atoms within the carbon framework to create Fe-SAzymes.^120^ Meanwhile, the immobilization of bioreceptors can be achieved by modifying the surface of Fe-SAzymes with amino-aldehyde compounds. Given that, in 2024, Wang's research team reported a dual-mode electrochemical and colorimetric technique for the simultaneous determination of multiple OPs pesticide residues in vegetables.^117^ As shown in Fig. 8b, Fe-SAzymes are developed through the Fe atoms incorporation into ZIF-8, taking advantage of the diverse boiling points of Fe and Zn during high-temperature pyrolysis. The Fe-SAzymes were surface-modified by attaching amino-aldehyde groups to facilitate the immobilization of the aptamer. These modified Fe-SAzymes exhibited peroxidase-like activity, resulting in the oxidation of TMB to form the blue oxidized product, TMBox. This reaction generated a colorimetric signal at approximately 452 nm under visible light. When the wide-spectrum aptamer interacted with OPs, a complex formed in close proximity to the nanozyme surface, influencing its catalytic function due to the toxic effects. Consequently, the concentration of OPs in the sample can be quantified based on this toxic impact. Furthermore, the complex containing Methylene Blue (MB) exhibited proximity to the chip interface because of a conformational alteration, which resulted in changes to the rate of ion migration and the potential of the electrode membrane. The OPs content can be evaluated by monitoring the alteration in potential. By leveraging their particular benefits, these two techniques mutually improved and validated the sensitivity and accuracy of the aptasensor with a LOD as low as 3.55 fM and a wide concentration range of 10^−13^ to 10^−2^ M, enabling the quantitative and qualitative identification of multiple OPs.

Single-atom metal–nitrogen–carbon (M–N–C) nanomaterials are considered a promising alternative to natural enzymes. However, their catalytic activity is still limited by certain constraints. Modifying the electronic and geometric structures of atomic active sites through the coexistence of atomic clusters and single atoms presents a promising approach for designing superior M–N–C catalysts. In this context, Zhao and coworkers introduced a model Fe–N–C catalyst, denoted as FeAC/FeSA-NC, which combines Fe single atoms with Fe atomic clusters on N-doped porous carbon.^121^ This catalyst was synthesized using a ligand-mediated approach involving the pyrolysis of Fe(ii)–phenanthroline complexes assembled within zeolitic-imidazolate-frameworks (ZIF-8@Fe-Phen). The FeAC/FeSA-NC catalyst prepared in this study reveals remarkable oxidase-like activity through the activation of oxygen into the reactive oxygen species, O_2_^−^ (superoxide radicals). DFT simulations prove that the interaction between iron clusters and single iron atoms results in a reduction in activation energy, thereby increasing the catalytic performance. As a concept application, the FeAC/FeSA-NC nanozyme is exploited for the ratiometric fluorescence assessment of OPs and acetylcholinesterase activity. This approach relies on the inhibitory effect of thiols on the enzymatic activity of the nanozyme. The established ratiometric nanoprobe for OPs identification fulfills a brilliant linearity over 0.005 to 50 ng mL^−1^, and a LOD as low as 1.9 pg mL^−1^. A brief description of the quality assurance parameters (*e.g.*, name of the SANzyme, LOD, and LDR) related to such studies is provided in Table 2. The LOD of SANzyme is typically lower than that of other nanozymes when detecting the same analyte. Pesticide residues can be detected more quickly and accurately in environmental samples, such as water, soil, and air, when using SANzyme compared to other nanozymes. Sensors based on SANzyme have also been employed for on-site detection of pesticide residues, providing a new method for real-time and online monitoring. Additionally, harnessing the catalytic properties of individual SANzyme molecules facilitates the degradation and immobilization of pesticide residues, offering innovative solutions for pollution control.

**Table 2:** Summary of various SANzyme-based biosensors used for the detection of pesticide residues

Name of the SANzyme	Target contaminant	Sensing substrate	LDR	LOD	Reference
Co_3_N@Co SACs	Imidacloprid	H_2_O_2_	0.05–10 ng mL^−1^	1.7 pg mL^−1^	80
SACe-N-C	Omethoate, methamidophos, carbofuran, and carbosulfan	AChE	—	55.83, 71.51, 81.81, and 74.98 ng mL^−1^, respectively	54
Ce-GONRs	Diafenthiuron and carbaryl	TMB	10–1500 ng mL^−1^ and 2–800 ng mL^−1^	0.57 and 0.23 ng mL^−1^	82
Ni-NPC	Carbamate	ATCh	—	1.5 ng mL^−1^	122
Ir(iii)/GO SAzymes	Pirimicarb	ATCh-H_2_O_2_	10–300 nM	2.81 nM	84
MOFs Fe_2_O_3_/MIL-100(Fe)	Carbendazim	H_2_O_2_	10 pg mL^−1^ to 50 ng mL^−1^	1.8 pg mL^−1^	86
Mn SAN	Acetamiprid	—	1.0–10 000 pg mL^−1^	0.3 pg mL^−1^	123
Fe–SAs/NC	—	H_2_O_2_	2 to 70 U L^−1^	0.56 U L^−1^	96
Fe–N–C SAzymes	Acetamiprid	TMB	—	16.9 nM	124
Apt-CSNLFA	Acetamiprid	—	1–150 ng mL^−1^	0.17 ng mL^−1^	103
Fe–N/C SAzymes	Organophosphorus	TMB	1–100 nM	0.4177 nM	114
Fe–N/C SAzymes	Malathion	TMB-H_2_O_2_	—	0.42 nM	115
Fe–N/C SAzymes	Chlorpyrifos	TMB-ATCh	0.05–10.0 μg mL^−1^	2.11 ng mL^−1^	116
Fe/CN-800-A	Chlorpyrifos	ATCh	1–50 ng mL^−1^	0.25 ng mL^−1^	118
SA-Fe-NZ	Organophosphorus	TMB	10^−13^ to 10^−2^ M	3.55 fM	117
Fe_AC_/Fe_SA_-NC	Organophosphorus	AChE	0.005–50 ng mL^−1^	1.9 pg mL^−1^	121

## 7. Conclusion and perspectives

In this study, we delineate the enzyme-like characteristics of SAzymes, emphasizing their SOD, POD, and CAT properties. The primary objective of this report is to discuss the application of SAzymes for monitoring various pesticide residues in agricultural food products, with a specific focus on organophosphate pesticides (OPPs), carbamates, acetamiprid, pyrethroids, and other pesticide classes. These studies serve as a proof-of-principle for exploring the expanding applications of SAzyme-based catalytic biosensors in monitoring pesticide residues. Our analysis was broadened to evaluate and compare the effectiveness of SAzymes based on various criteria, such as LOD, linear range, and recovery range. Although there have been significant advancements in the synthesis, design, and application of single-atom nanozymes, further research is necessary to deepen our understanding and advance innovative single-atom nanozymes for sensitive biosensing. In this discussion, we will examine various challenges and opportunities related to mechanisms, materials, and applications.

In comparison to natural enzymes, the inherent catalytic efficiency of SAzymes, which includes both activity and selectivity, does not meet the expected levels. SAzymes typically exhibit limited selectivity and possess multienzyme-like function, which renders them susceptible to interference from analogs with high background levels (for example, the detection of H_2_O_2_ may be compromised by O_2_). To address this issue, it is advisable to modulate the electronic structure of SAzymes by emulating various aspects of natural enzymes, including electronic and geometric characteristics such as coordination environments and oxidation states. Additionally, the implementation of theoretical calculations to facilitate the rapid screening of enzymatic activity is recommended. Furthermore, the synergistic effects arising from the interaction of various single atoms, their supports, or doping strategies present significant opportunities for enhancing the catalytic performance of SAzymes. It is essential to modify SAzymes by incorporating specific functional groups to enhance their biocompatibility and hydrophilicity, in order to ensure the desired properties of stability and selectivity. Clarification of the catalytic mechanism is important for the design of superior SAzymes comprising stability, specificity/selectivity, and catalytic activity. Particularly, the application of DFT, recognized as an effective approach for calculating and estimating the descriptors that affect catalytic activity, holds great importance for the investigation and prediction of catalytic behavior and the associated mechanisms. However, it remains very complex and expensive to use DFT for a substantial number of chemical interactions, and the estimate of the catalytic function of SAzymes is far more challenging than the validation of experimental outcomes through DFT, especially in terms of model construction. Machine learning is an effective and attractive device to support DFT in calculating the structure–activity correlation of SAzymes. Despite significant advancements in the application of SAzymes within the biological domain, their development remains in the early stages. For instance, in the biosensing field, it is confined to the analysis of restricted targets and the diagnosis strategies are somewhat monotonous (electrochemical, fluorescence, and colorimetric assays). Thus, other output modes like photoelectrochemical sensing, and surface-enhanced Raman must be extended. In order to address this issue, it is essential to develop bifunctional SAzymes that integrate the characteristics of other functional materials. From a materials perspective, the precise regulation of active sites at the atomic scale presents significant challenges. Initially, active metal atoms tend to self-assemble, leading to the formation of nanocrystals during the synthesis process. Although most nanocrystals can be removed through acid washing, complete elimination is not always feasible, and certain all-metal-based materials cannot employ this method. Moreover, realizing the accurate regulation of SAzymes by uniformly separated single-atom sites remains an issue. The same concern arises when modifying SAzymes with targeted biomacromolecules and functional groups, as well as achieving well-controlled parameters such as size, shape, stability, charge, and surface chemistry. Designing and synthesizing appropriate structures of SAzymes presents a significant challenge in order to achieve optimal performance for applications in environmental protection, food safety, and biomedical research.

We believe there is significant potential for interdisciplinary research in the field of SAzyme applications, particularly in medicine, biology, electrical engineering, mechanical engineering, and materials science. For instance, integrating SAzyme with 3D printing technology facilitates the development of wearable electronics and portable point-of-care biosensors for continuous closed-loop monitoring. Nevertheless, we contend that further research is essential before considering commercial utilization. It is crucial to verify the long-term durability and stability of SAzyme-based systems before they can be practically implemented and commercialized.

## Data availability

No new data were created or used for the research described in the article.

## Conflicts of interest

The authors declare that they have no known competing financial interests or personal relationships that could have appeared to influence the work reported in this paper.
